# Note by a Surgeon on Active Service

**Published:** 1915-08-14

**Authors:** 


### Note by a Surgeon on Active Service.

**Protection of the Abdomen on the March.**

"Next to the head, it is the abdomen which especially requires protection. This, in our Service, is well afforded by the flannel shirt and drawers worn by the soldier; and hence recourse to the abdominal bandage, held in such high esteem in the British and French Colonial Services, is unnecessary. However desirable such an additional protection may be in times of cholera or diarrhœal epidemic, the value of its general use in the tropics may seriously be questioned. To march on a hot day with such a band over the abdomen is evidently illogical, since it increases the temperature and weakens the powers of resistance, thus perhaps even predisposing to the diarrhœa which it is supposed to prevent. The native West Indian troops of the British Army wear a species of Zouave uniform, the trousers of which are cut unusually high in the waist, and so afford an excellent protection to the abdomen."—*Munson, in "Manual of Military Hygiene."*

